# The effect of social determinants of health on quality of life among Afghan refugees in Iran during the COVID-19 pandemic: a path analysis

**DOI:** 10.1186/s12888-022-04502-0

**Published:** 2023-01-04

**Authors:** Zohreh Mahmoodi, Giti Bahrami, Nooshin Ghavidel, Hamed Seddighi

**Affiliations:** 1 Social Determinants of Health Research Center, Alborz University of Medical Sciences, Karaj, Iran; 2 Non-Communicable Diseases Research Center, Alborz University of Medical Sciences, Karaj, Iran; 3 Department of Psychology, University of Groningen, Groningen, The Netherlands; 4 Unit of Child and Family Welfare, Department of Pedagogical and Educational Sciences, University of Groningen, Groningen, The Netherlands

**Keywords:** COVID-19, DASS-21, Posttraumatic stress symptoms, Quality of life, Social determinants of health, Self-rated health

## Abstract

**Background:**

The COVID-19 pandemic has created significant challenges in 2020 in the world and Iran. To help vulnerable groups such as refugees during the response and recovery phases of the COVID-19 pandemic, identifying the quality of life (QOL) and its associated factors is helpful. Considering that research in this field is limited, this study evaluated the effect of social determinants of health on the quality of life among Afghan refugees in Iran during the COVID-19 pandemic.

**Methods:**

We conducted a cross-sectional study on 300 Afghan refugees and migrants in Alborz province, Iran, from February to May 2022 using Convenience sampling. Data were completed using the socioeconomic status scale (SES), World Health Organization's quality of life -BREF (WHOQOL), Depression, Anxiety and Stress Scale—21 Items (DASS-21), and COVID-19 Post-Traumatic Stress Disorder Checklist (COVID-PTSD). In addition, path analysis was applied to evaluate the relationships among the research variables with quality of life.

**Results:**

64.3% of the study participants were male, with a mean of 29.29 ± 9.64 years. The path analysis showed that SES had the most positive relationship (B = .266), and the number of COVID-19 cases had the most negative relationship (B = -.169) with the quality of life from both paths. The self-rated health had the most positive relationship (B = .25), and the DASS score had the most negative relationship (B = -.2) with the quality of life through only one path. Access to medical services was the only variable that indirectly had a positive causal relationship with QOL (B = .044).

**Conclusion:**

We provided an empirical model that illustrates the relationships between quality of life and social determinants of health among Afghan refugees and migrants during the Covid19 pandemic. The negative emotional states of Depression, Anxiety, and Stress (DAS) as a mediator play an essential role in the quality of life and other variables.

## Introduction

Conflicts, wars, disasters triggered by natural hazards, and climate change may threaten all generations, causing many people to migrate from treacherous, vulnerable, and stressful conditions to destination countries [1, 2]. Refugees might suffer exploitation, prejudice, and violence during their journey and stay in host countries, which may negatively affect their health [3]. Due to its geographical location, ethnic structure, and internal unrest, Afghanistan continuously generates immigrants, and Afghans have been migrating to neighbouring countries such as Iran for centuries [4, 5].

According to the United Nations High Commissioner for Refugees (UNHCR), The Islamic Republic of Iran hosts one of the largest and most protracted urban refugee populations worldwide, with approximately 3 million Afghans living in the country [6]. The challenges of Afghan refugees in Iran include a low level of education, low health literacy, a lack of insurance, low access to healthcare services, and high medical expenses [7]. In addition, evidence showed that the prevalence of communicable and non-communicable disorders, psychological problems, and mental disorders among Afghan refugees in Iran is higher than in the Iranian population [7, 8].

The COVID-19 pandemic has created significant challenges in 2020 in the world and Iran. Studies have reported psychological problems, such as anxiety, depression, and Post-Traumatic Stress Disorder (PTSD). Refugees may be significantly affected by COVID-19 as the pandemic reminds them of their past conflicts and traumas of persecution [9, 10]. Results of scientific research have shown that several factors are associated with the prevalence and spread of COVID-19 disease, including age, very high contact, poor general health, nutritional status, underlying chronic conditions [11, 12], as well as psychological problems such as depression, anxiety [13]. Most refugees may live in crowded living conditions, making it difficult to control the COVID-19 outbreaks in these groups [14–16]. Studies have revealed that the health needs of refugees have been largely neglected in global healthcare responses [17], health inequalities have increased, and access to health services has often significantly been restricted among Afghan migrants during the pandemic [18].

Quality of life (QOL) is defined as an 'Individual' perception of their position in life in the context of the culture and value systems in which they live and concerning their goals, expectations, standards and concerns [19]. A wide range of factors was found to have significant associations with QOL, including mental health, higher age, adverse life events, post-migration living problems (PMLP), socioeconomic living conditions, and socio-religious aspects [20, 21]. In addition, studies have indicated that refugees' QOL is highly influenced by the conditions they live in post-migration and social determinants of health; mental health mediates these effects [22, 23].

To help vulnerable groups such as refugees during the COVID-19 pandemic, identifying the QOL and its associated factors will help inform governments on how to address the challenges of health emergencies better. However, to our best knowledge, there is no research to examine social determinants of health factors, including socioeconomic status, access to healthcare services, and mental health in the quality of life together in Afghan refugees during different phases of the COVID-19 pandemic, such as response and recovery phases. Therefore, considering the challenges of the COVID-19 pandemic and the critical role of social determinants of health, we examined the effect of social determinants of health on the quality of life among Afghan refugees in Iran during the COVID-19 pandemic.

## Materials and methods

### Design and participants

This study cross-sectional study was conducted on 300 Afghan refugees and migrants in Alborz province, Iran, from February to May 2022 by using convenience sampling. The inclusion criteria were age over 15 years, Afghan nationals who have lived in Iran for at least one year, ability to answer questions, and willingness to participate in the study. Exclusion criteria were mental retardation and dementia/Alzheimer's disease. The samples were selected from the general population by using convenience sampling. After selecting the subjects, trained experts collected data through interviews and questionnaires. This research was performed by the latest version of the Declaration of Helsinki and with the approval of the research ethics committee of Alborz University of Medical Sciences (IR.ABZUMS.REC.1400.337). Informed consent was obtained from all participants after being informed of the nature of the research.

### Data collection

Data were completed using a questionnaire including:

1. A socio-demographic checklist included age, gender, education level, marital status, occupation, income, number of family members, the history of underlying chronic disease, history of COVID-19 disease, and the frequency of infection with COVID-19.
2. Socioeconomic status scale (SES): SES consisted of 6 questions, including education, income, economic class, and housing status, which are scored based on a Likert scale from 1 to 5, and a total score ranging from 6 to 30. Validity and reliability have been performed in Iran [24].
3. The World Health Organization's quality of life -BREF (WHOQOL) [25] was used to determine the quality of life in participants. The questionnaire consists of 26 self-report questions measured on a 5-point Likert scale (1 = Very poor or never, and 5 = Very good or always). The first two items were overall Quality of Life (QOL) and Overall Health Status (OHS). The remaining 24 items encompass four domains: physical health, psychological health, social relationships, and environmental. According to the manual, scores were transferred to a scale from zero (very poor) to 100 (excellent). We considered the total QOL score for path analysis, in which a higher score indicates a better quality of life. The questionnaire has been validated in an adult Iranian population [26].
4. Depression, Anxiety and Stress Scale—21 Items (DASS-21) questionnaire [27]: we used the Persian version of DASS-21 to assess the depression, anxiety and stress (DAS), which has reliability and validity [28]. The DASS-21 is a self-report questionnaire that includes 21 items, seven items for each category. Patients rated each item on a scale from 0 (did not apply to me) to 3 (applied to me very much). Total scores were calculated by summing the item scores in each sub-scale. Therefore, total scores for the DASS-21 scale range from 0 to 63. We considered the total DASS-21 score for path analysis.
5. The COVID-19 Post-Traumatic Stress Disorder Checklist (8 questions): The PTSD 8-item Inventory [29] is derived from the HTQ (Harvard Trauma Questionnaire) part IV [30] and is a short PTSD scale that includes eight items measuring Post-traumatic stress symptoms. Each item is presented on a 4-point Likert scale (from 1 = not at all to 4 = very often). PTSD-8 consists of three clusters, including intrusion, avoidance, and hypervigilance. PTSD and subscales score was calculated by summing the scores of the questions. Possible PTSD is defined by at least 1 item within each PTSD symptom cluster (intrusion, avoidance, hypervigilance) with a score of 3 or higher. PTSD-8 has good psychometric properties. The validity and reliability of the PTSD 8-item Inventory have been confirmed by Hansen et al. [29]. The questionnaire has also been validated in the Iranian population during the COVID-19 pandemic [31].
6. Access to healthcare services: This checklist includes three questions about the distance from home to healthcare centres, travel costs to healthcare centres, and waiting time to receive services ranging from 3 to 12. The lower the score shows, the better the access to health services.

Self-rated health includes one question about people's perceptions of their health, ranging from 1 to 4 (bad, average, good, and excellent).

### Research variables

Variables used in the path analysis included age, the number of family members, Socioeconomic status, number of COVID-19 conflicts, access to medical services, self-rated health, DASS, PTSD, and quality of life.

### Statistical analysis

All analyses were performed using SPSS 22.0. First, the study population's demographic characteristics were described using descriptive statistics, mean, Standard Deviation (SD) for continuous variables, and frequency (%) for categorical variables. Next, the Pearson correlation coefficient was calculated to evaluate the relationships among the variables. Then, Path analysis was applied to evaluate the relationships among the research variables mentioned above. Path analysis is an extension of the regression model, which assesses the effects of a set of variables acting on a dependent variable via multiple causal pathways [32]. The model fit is acceptable with a cutoff value of 0.9 for the comparative fit index (CFI), Goodness of fit index (GFI), and Bentler-Bonett Normed fit index (NFI), as well as a cutoff value of < 0.05 for the root, mean squared error of approximation (RMSEA) [33].

**Fig. 1:**
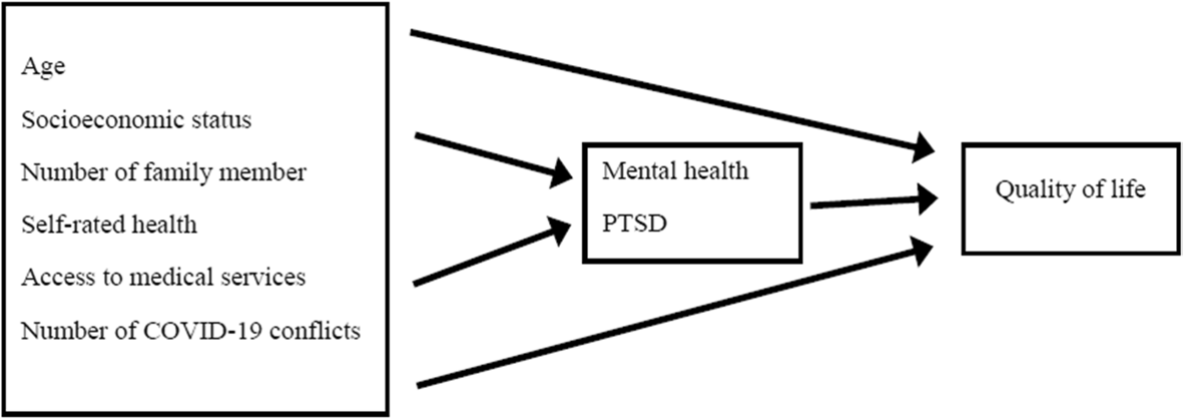
Theoretical, conceptual framework of the relationships among the study variables

According to previous studies [10, 20–23, 34, 35], we developed a theoretical, conceptual framework using Path analysis (Fig. 1).

## Results

A total of 316 respondents completed our questionnaire. In the end, 16 were excluded because they were below 15 years or had incomplete questionnaires. Of the 300 participants in the study, 64.3% were male. The mean ± SD age of people was 29.29 ± 9.64. The most educational level was elementary (32.3%) and illiterate (29.7%); 64% were workers. In addition, 60 (20%) participants had at least one underlying disease. The most frequent disease was diabetes (14 = 4.7%). The demographic characteristics of patients are shown in Table 1.

**Table 1:** Sociodemographic characteristics of subjects

**Variables**		**No. (%)**
Sex	Male	120 (39%)
	Female	188 (61%)
Educational level	Illiterate	89 (29.7%)
	Elementary	97 (32.3%)
	< Diploma	62 (20.6%)
	≥ Diploma	52 (17.4%)
Income, Rial	≤ 50,000,000	217 (72.3%)
	> 50,000,000	83 (27.7%)
Marital status	Single	120 (40.0%)
	Married	180 (60.0%)
Underlying disease	Yes	60 (20.0%)
Infected with COVID-19	yes	141 (47.0%)

The mean ± SD of SES, COVID-PTSD and DASS were 9.43 ± 3.5, 14.75 ± 3.7, and 20.67 ± 9.9, respectively. As well as the mean ± SD scores of QOL subscales, including physical health, psychological health, social relationships, and environmental domain, were 51.62 ± 13.64, 46.14 ± 18.14, 48.05 ± 22.76, and 39.60 ± 14.66, respectively.

Before path analysis, bivariate analysis was used to assess the correlations between variables. QOL was directly correlated with the number of family members, SES, self-rated health, and access to medical services and inversely correlated with the number of COVID-19 conflicts, PTSD, and DASS (Table 2).

**Table 2:** Correlations between structural parameters

Variables	Age	NF	COVID	SES	SRH	AMS	PTSD	DASS	QOL
Age	**1**								
NF	.216^***^	1							
COVID	.043	-.181^**^	1						
SES	.012	.195^**^	-.148^*^	1					
SRH	-.065	.248^***^	-.112	.153^**^	1				
AMS	.087	.002	-.137^*^	-.062	-.009	1			
PTSD	-.087	-.145^*^	.206^***^	.091	-.039	-.246^***^	1		
DASS	.052	-.076	.107	.197^**^	-.046	-.234^***^	.558^***^	1	
QOL	-.039	.277^***^	-.224^***^	.356^***^	.378^***^	.141^*^	-.159^**^	-.195^**^	1

*NF* Number of family members, *COVID* Number of COVID-19 conflicts, *SES* Socio-economic status, *SRH* Self-rated health, *AMS* Access to medical services, *PTSD* post-traumatic stress disorder, *DASS* Depression, Anxiety and Stress Scale, *QOL* Quality of life

**Fig. 2:**
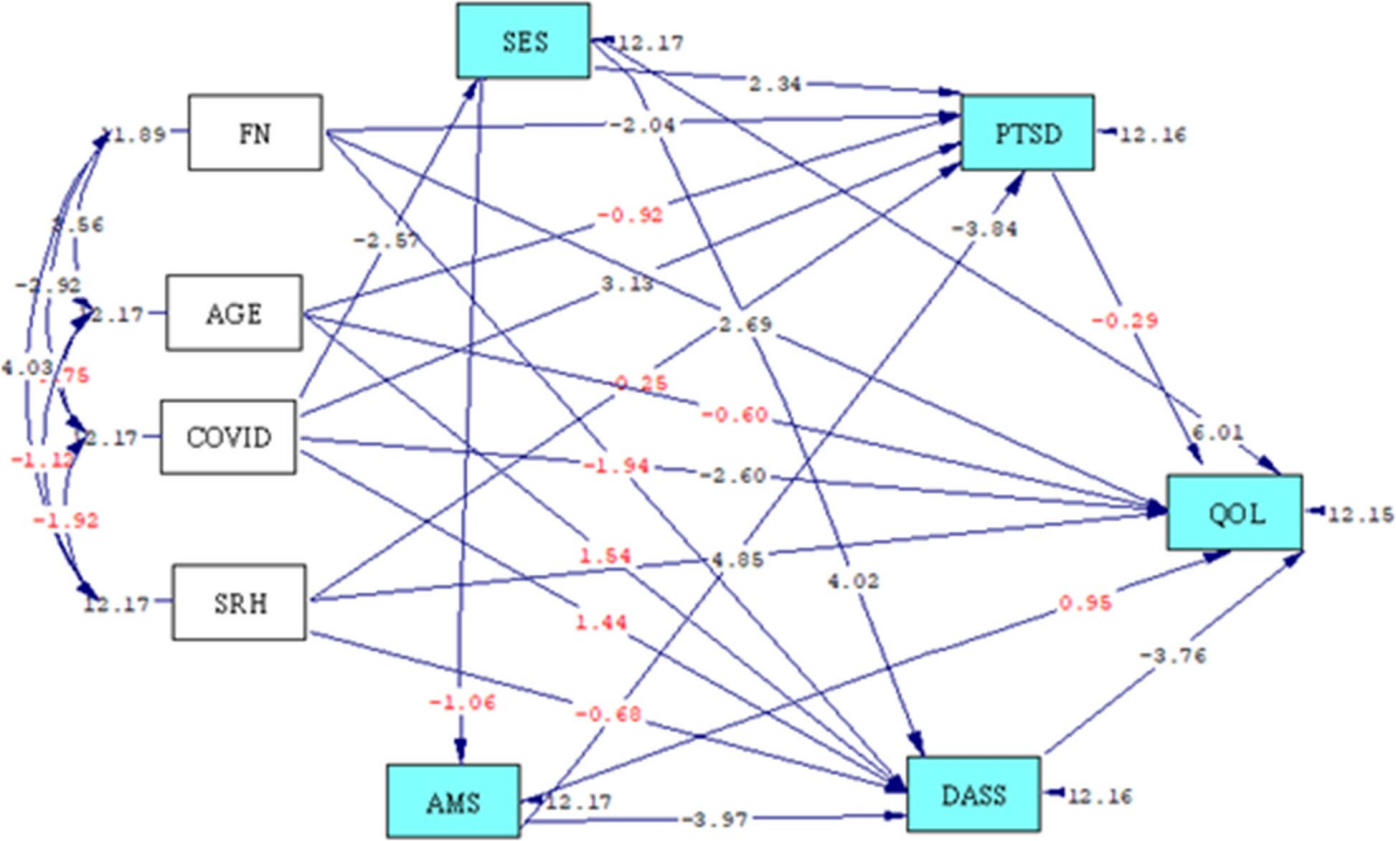
Full Empirical Model (Empirical Path Model between Quality of Life and Social Determinants of Health) according to T-value. (Black color: T-value > 1.96 is significant; Red color: T-value < 1.96). SES = Socio-economic status, FN = Family number, COVID = Number of COVID-19 conflicts, SRH = Self-rated health, AMS = Access to medical services, DASS = Depression, Anxiety and Stress Scale, QOL = Quality of life

The relationship between QOL and social determinants of health in Afghan refugees was studied based on the path analysis model. Figure 2 shows the full empirical path model between the QOL and social determinants of health according to T-value. T-value > 1.96 is significant and is shown in black colour.

**Fig. 3:**
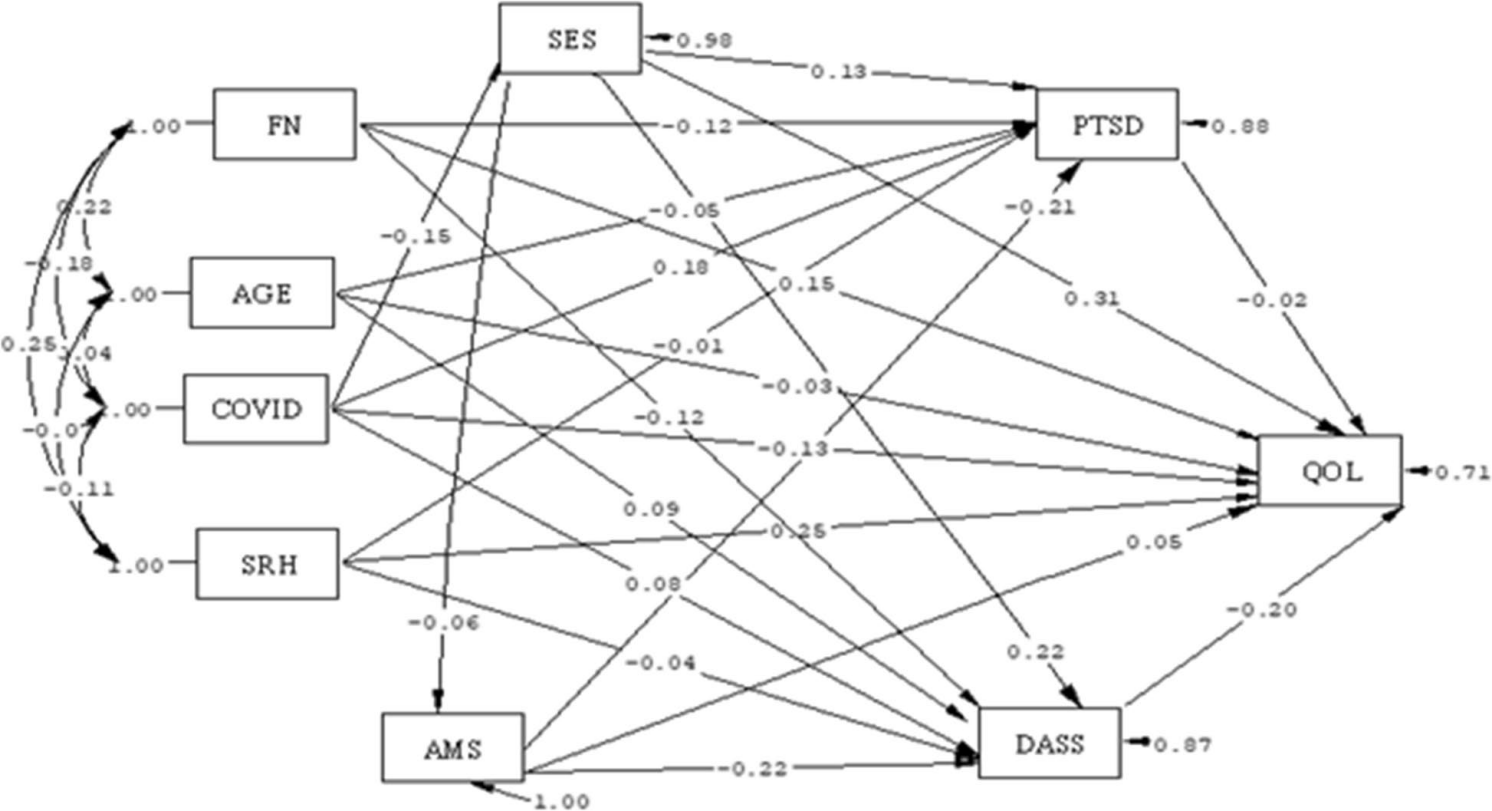
Full Empirical Model (Empirical Path Model between Quality of Life and Social Determinants of Health) according to Standard B. SES = Socio-economic status, FN = Family number, COVID = Number of COVID-19 conflicts, SRH = Self-rated health, AMS = Access to medical services, DASS = Depression, Anxiety and Stress Scale, QOL = Quality of life

Based on the results of the path analysis, among the variables that had a causal and significant relationship with the QOL from only one path, self-rated health (SRH) had the most positive relationship (B = 0.25), and the DASS score had the most negative relationship (B = -0.2) with the QOL. In other words, an increase in the SRH score increases the quality of life score, and an increase in the DASS (mental disorder) score decreases the quality of life score. Access to medical services was the only variable that indirectly had a positive causal relationship with QOL (B = 0.044), so an increase in the AMS score increases the quality of life score. The number of family members directly had a positive causal relationship with QOL (B = 0.15). Among the variables that had a significant causal relationship with QOL from both paths, socioeconomic status had the most positive relationship (B = 0.266), and the number of COVID-19 conflicts had the most negative relationship (B = -0.169). In other words, an increase in socioeconomic status increases the quality of life score, and an increase in the number of COVID-19 conflicts decreases the quality of life score. (Fig. 3 and Table 3). Figure 3 shows the full empirical path model between the QOL and social determinants of health according to standard B.

**Table 3:** The direct and indirect effects on QOL and social determinants of health

Variables	Standard B			Unstandardized β			R2
	Direct effect	Indirect effect	Total effect	Direct effect	Indirect effect	Total effect	
**Age**	-.03	-.017	-.047	-.04	-.0198	.059	.46
**NF**	.15^*^	.0264	.15^*^	.84^*^	.13	.84^*^	
**COVID**	-.13^*^	-.039^*^	-.169^*^	-1.49^*^	-.431^*^	-1.92^*^	
**SES**	.31^*^	-.044^*^	.266^*^	1.05^*^	-.15^*^	.9^*^	
**SRH**	.25^*^	.0082	.25^*^	3.31^*^	.104^*^	3.41^*^	
**AMS**	.05	.044^*^	.044^*^	.23	.2^*^	.2^*^	
**DASS**	-.2^*^	-	-.2^*^	-.26^*^	-	-.26^*^	
**PTSD**	-.02	-	-.02	-.05	-	-.05	

*NF* Number of family members, *COVID* Number of COVID-19 conflicts, *SES* Socio-economic status, *SRH* Self-rated health, *AMS* Access to medical services, *PTSD* post-traumatic stress disorder, *DASS* Depression, Anxiety and Stress Scale, *QOL* Quality of life

^*^Statistically significant

The model fit results indicated the desirability, high suitability, and reasonableness of the adjusted relationships of the variables in the model. (Table 4).

**Table 4:** Goodness of fit of empirical path model among quality of life and social determinants of health

Fit Index	X^2^	df	X^2^/df	CFI	GFI	NFI	RMSEA
Model Index	28.47	6	4.74	.97	.98	.095	.048
Acceptable range	X2/df < 5			> .9	> .9	> .9	< .05

*CFI* (comparative fit index), *GFI* (Goodness of fit index), *NFI* (Bentler-Bonett Normed fit index), *RMSEA* (root mean squared error of approximation)

## Discussion

In this study, we evaluated the association between quality of life with social determinants of health, including sociodemographic factors, SES, DAS, PTSD, access to medical services, self-rated health, and the number of COVID-19 conflicts among Afghan refugees and migrants during the COVID-19 pandemic trough path analysis.

We observed DASS score had a negative relationship with quality of life only through the direct path. These findings extend to studies [21, 34, 36], confirming that those affected by anxiety and depression had unfavourable QOL. In line with our study, a study in Turkey investigated sociodemographic factors and the mental health of women refugees on quality of life (QOL) and found that mental health mediates these effects [23]. People with mental problems experience a poor quality of life due to distress, hopelessness, and demoralization; lack of control, choice, and autonomy; low self-esteem and confidence; feeling of not being part of society; and reduced activity [37].

The current study's findings showed that self-rated health (SRH) had a positive relationship with quality of life only through a direct path consistent with the study of Pitkala et al. [38]. Physical health is essential for mental health because physical illness may cause anxiety and isolation [39].

In this study, access to medical services was the only variable that indirectly had a positive causal relationship with quality of life, consistent with another study [39, 40] that showed relationships between access to health services and mental health and quality of life. The general quality of life, however, includes an individual's evaluation of all aspects of life, including factors such as the safety of the environment in which they live, whether they have access to health care and social services, and their current spiritual status [41].

Socioeconomic status positively correlated with the quality of life on both paths. In line with our survey, other studies have shown that high levels of psychological distress were most common among individuals with lower levels of SES [39, 42]. Also a study showed an indirect effect of SES on the quality of life by path analysis [43].

We observed that number of COVID-19 conflicts negatively affected the quality of life from both paths. A study on refugees in Bangladesh also showed a negative impact of the COVID-19 pandemic on their quality of life [44]. The mean PTSD of Afghan refugees in our study was higher than a study of Iranian pupations [45], which may be because of a higher level of stress, anxiety, depression, and other immigration problems in Afghan refugees. A qualitative study in Iran illustrated that Afghan refugees, especially women, are very vulnerable to COVID-19. The reasons include their little knowledge and information about COVID-19, limited access to information resources, family challenges (intensified experience of violence and conflict in the family, problems related to childbirth and pregnancy), socioeconomic challenges (exacerbation of economic problems, high-risk living conditions, social isolation, limited support of social and health organizations), health issues (problems related to treatment, injustice in providing services and facilities) and problems after the death of a COVID-19 patient (burial challenges for immigrants; lack of funeral rites) [46].

In our study, the number of family members had a positive causal relationship with quality of life. When family members are together and have a good relationship, they live better together, which can help increase their quality of life. Therefore, it can be expected that family cohesion is the determining factor in people's quality of life during the pandemic period [47].

The findings of our study showed that negative emotional states of depression, anxiety and stress (DAS) are one of the most critical factors affecting the quality of life of Afghan refugees. Psychosocial support during the pandemic is one of the things emphasized by humanitarian organizations [48]. In addition, the intersectionality of social determinants of health, such as age, gender, ethnicity, income status, etc., increases the need for these supports [49]. According to a report by the Women's Commission for Refugee Women and Children, "Too often invisible, too often forgotten, and too often overlooked, refugees with disabilities are among the most isolated, socially excluded, and marginalized of all displaced populations" [50]. Intersectionality in the group of refugees makes them more vulnerable and needs psychosocial support.

Limitation: all the questionnaires used in the current research were self-assessments. Therefore, self-report bias due to personal attitudes was inevitable.

## Conclusion

We provided an empirical model that illustrates the relationships between quality of life and social determinants of health, including sociodemographic factors, SES, DAS, PTSD, access to medical services, self-rated health, and the number of COVID-19 conflicts among Afghan refugees and migrants. Variables that affect QOL only through a direct path included mental disorders, which had a negative relationship, self-rated health (SRH), and the number of family members who had a positive relationship with quality of life. Socioeconomic status had the most positive, and the number of COVID-19 conflicts had the most negative relationship with the quality of life from both direct and indirect paths. Access to medical services was the only variable that indirectly had a positive causal relationship with quality of life. Negative emotional states of DAS as a mediator play an essential role in the quality of life and other variables, so public health policymakers should pay more attention to the mental health of Afghan refugees to improve their quality of life.

## Data Availability

The datasets used during the current study available from the corresponding author on reasonable request.
